# Sorptive Uptake Studies of an Aryl-Arsenical with Iron Oxide Composites on an Activated Carbon Support

**DOI:** 10.3390/ma7031880

**Published:** 2014-03-05

**Authors:** Jae H. Kwon, Lee D. Wilson, Ramaswami Sammynaiken

**Affiliations:** 1 Department of Chemistry, University of Saskatchewan, 110 Science Place, Saskatoon, SK S7N 5C9, Canada; E-Mail: jak878@mail.usask.ca; 2 Saskatchewan Structural Science Centre, University of Saskatchewan, 110 Science Place, Saskatoon, SK S7N 5C9, Canada; E-Mail: r.sammynaiken@usask.ca

**Keywords:** roxarsone, aryl-arsenical, magnetite, goethite, activated carbon, adsorption

## Abstract

Sorption uptake kinetics and equilibrium studies for 4-hydroxy-3-nitrobenzene arsonic acid (roxarsone) was evaluated with synthetic magnetite (Mag-P), commercial magnetite (Mag-C), magnetite 10%, 19%, and 32% composite material (CM-10, -19, -32) that contains granular activated carbon (GAC), and synthetic goethite at pH 7.00 in water at 21 °C for 24 h. GAC showed the highest sorptive removal of roxarsone and the relative uptake for each sorbent material with roxarsone are listed in descending order as follows: GAC (471 mg/g) > goethite (418 mg/g) > CM-10 (377 mg/g) CM-19 (254 mg/g) > CM-32 (227 mg/g) > Mag-P (132 mg/g) > Mag-C (29.5 mg/g). The As (V) moiety of roxarsone is adsorbed onto the surface of the iron oxide/oxyhydrate and is inferred as inner-sphere surface complexes; monodentate-mononuclear, bidentate-mononuclear, and bidentate-binuclear depending on the protolytic speciation of roxarsone. The phenyl ring of roxarsone provides the primary driving force for the sorptive interaction with the graphene surface of GAC and its composites. Thus, magnetite composites are proposed as multi-purpose adsorbents for the co-removal of inorganic and organic arsenicals due to the presence of graphenic and iron oxide active adsorption sites.

## 1. Introduction

**Figure 1.**
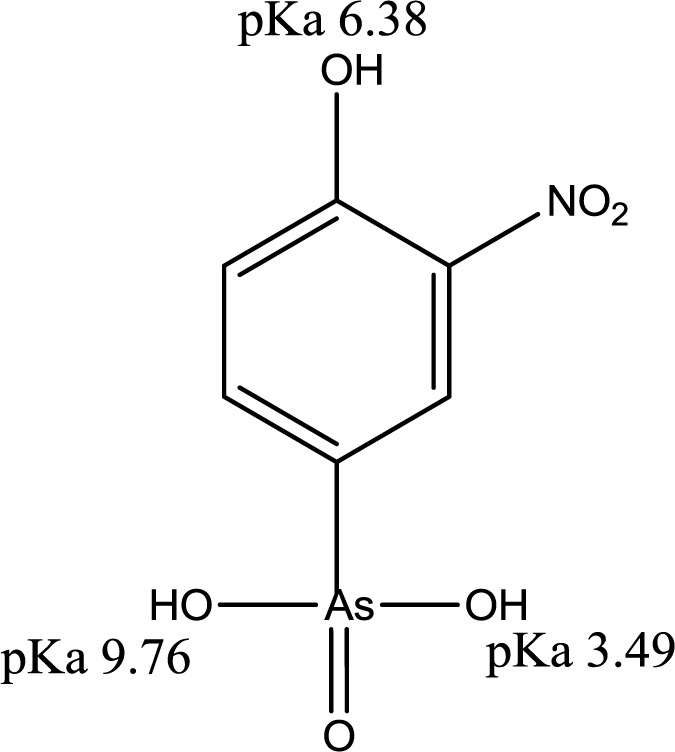
Molecular structure of roxarsone and its pK_a_ values in aqueous solution.

Arsenic species occur naturally in surface water such as rivers, lakes, reservoirs, and ponds by the natural processes of soil erosion, mineral leaching, volcanic deposits, and geochemical weathering processes [1]. Anthropogenic inputs to the atmosphere through mineral smelting operations, fossil-fuel combustion, and consumption of organo-arsenicals by poultry contribute to the overall fate and distribution of arsenic: geological and anthropogenic activities (e.g., (CH_3_)_3_As, As_4_O_6(s)_, As_4_O_10(s)_) → water pollution → soil and sediments → bioaccumulation [2]. Arsenic has variable oxidation states in the environment (3−, 0, 3+, and 5+), particularly in aquatic environments where the speciation depends on the relative redox potential and the pH conditions [3]. Common occurring forms of arsenic are its oxyanions (arsenite (As^3+^) or arsenate (As^5+^)) [3], where the latter is the most thermodynamically stable form in surface water environments. Arsenite is relatively stable under mild reducing conditions in anoxic ground waters and is considered the more thermodynamically stable form [3]. In most natural waters, arsenic (III) occurs as the non-ionized form of arsenous acid (H_3_AsO_3_, pK_a_ = 9.22) and may interact weakly with most solid surfaces [4]. Depending on the pH and oxidizing conditions, the efficient isolation of various protolytic forms of arsenic (III) species with traditional treatment methods such as adsorption, precipitation, *etc.*, represent a technical challenge [4]. Roxarsone (4-hydroxy-3-nitrobenzene arsonic acid) shown in Figure 1 is an aryl-arsenical feed additive for swine and poultry which promotes weight and serves as an anti-microbial agent. Roxarsone may contaminate soil and surface water supplies through the uncontrolled use of poultry litter as a fertilizer additive [5,6]. Roxarsone partially degrades via metabolic pathways in poultry and in soil environments which may yield more toxic inorganic forms of arsenic (*i.e.*, arsenite and arsenate) [7–10].

Arsenic contamination of drinking water has been highlighted because of its toxicity and variable occurrence [11]. Several countries such as Bangladesh, New Zealand, USA, Italy, and Malaysia are facing serious water security due to high-arsenic levels in their source drinking water supplies [12–14]. The World Health Organization (WHO) has established international health standards for arsenic in drinking water at <10 ppb to minimize the risks of arsenic exposure. The importance of arsenic in wastewater was recognized in Canada by the establishment of guidelines in the mining industry, as evidenced by the Metal Mining Effluent Regulations (MMER) [15]. As of 2010, Canadian mining industries are required to adhere to the release limits on various species; arsenic, copper, cyanide, lead, nickel, zinc, radium-226, and total suspended solids. In northern Saskatchewan, reports indicate that metals such as As, Mo, Ni and Se are found in the liquid and solid tailings of various mine sites [16].

Activated carbon is a microporous amorphous material with relatively high surface areas and surface functional groups with heteroatoms such carbonyl (C=O), hydroxyl (–OH), amino (–NH_2_), and thiol (–SH), depending on the oxidizing conditions [17]. These functional groups may serve as electron donors (Lewis base) and may contribute to form metal-pi interactions with metal cations (Lewis acid) on the surface of granular activated carbon (GAC) and on its basal planes. The pores of GAC offer surface adsorption sites which may also serve as a template site for the growth of magnetite in the case of supported composite materials [18].

Arsenic removal may employ various technologies: aeration, chlorination, sedimentation, precipitation/co-precipitation, adsorption, ion exchange, membrane separation including microfiltration, reverse osmosis, electrodialysis, ultrafiltration, and nanofiltration, and biological processes. Among the various methods, adsorption is a versatile method because of its relatively low cost and applicability to a wide range of waterborne contaminants depending on their chemical nature [12–14]. Recent studies have illustrated the utility of biomaterial-based adsorbents for arsenate anion removal in aqueous solution [19,20]. Similarly, iron oxide-based materials and their composites are versatile adsorbents due to the high affinity of such materials to inorganic arsenicals [12,21–23]. For example, the removal of metal cations and their oxyanions from wastewater employs iron oxides and oxyhydroxides (e.g., magnetite, Fe_3_O_4_; maghemite, γ-Fe_2_O_3_; hematite, α-Fe_2_O_3_; goethite; α-FeOOH), and aluminum oxides/oxyhydroxides (e.g., activated alumina, γ-Al_2_O_3_ and gibbsite, Al(OH)_3_). Such types of inorganic adsorbents have been used for decades due to their low cost and relatively high affinity toward inorganic arsenicals [21–23]. One limitation of such inorganic adsorbents is the potential leaching of the framework. Magnetite-graphene or magnetite-zeolite composites are examples of magnetite-based nanomaterials [22,23] which may release iron species due to the high *surface-to-volume* ratio of such sorbents. Thus, there is a need to investigate adsorbents with favourable binding affinity toward arsenicals whilst minimizing leaching of the framework during adsorptive processes.

The removal of roxarsone and its inorganic degradation products requires a multi-purpose adsorbent material with potential dual binding affinity toward inorganic and organo-arsenicals whilst minimizing the aforementioned leaching problems. We report an adsorption study of roxarsone in aqueous solution with composite materials containing activated carbon and iron oxide at variable composition. The adsorptive properties of the composites are compared with activated carbon (GAC) and two types of iron oxides (*i.e*., magnetite and goethite) with roxarsone. Although roxarsone may undergo chemical decomposition when used as a feed additive, it remains largely unmetabolized (~80%) as an organo-arsenical [7–10]. Therefore, the study herein is focused on the equilibrium and kinetic sorption properties of various adsorbent materials with roxarsone in aqueous solution.

## 2. Results and Discussion

### 2.1. Sorption Isotherms

**Figure 2.**
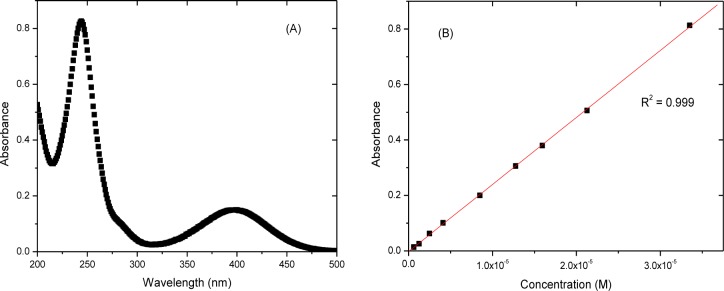
UV-Visible absorption spectrum of roxarsone (**A**) and a Beer-Lambert calibration curve (**B**) at pH 7.00 in water (10 mM) at 244 nm.

Calibration curves of roxarsone (Figure 2) were obtained using UV-vis absorption spectroscopy. The molar absorptivity (ε) of roxarsone at pH 7.00 was 27.3 × 10^3^ M^−1^cm^−1^ at λ = 244 nm, in agreement with an independent estimate for roxarsone (ε = 22.9 × 10^3^ M^−1^cm^−1^) at λ = 224 nm [24]. The spectral bands at 400 nm and 244 nm arise from electronic transitions of S_o_ → S_1_ (n-π*) and of S_o_ → S_2_ (π-π*), respectively.

**Figure 3.**
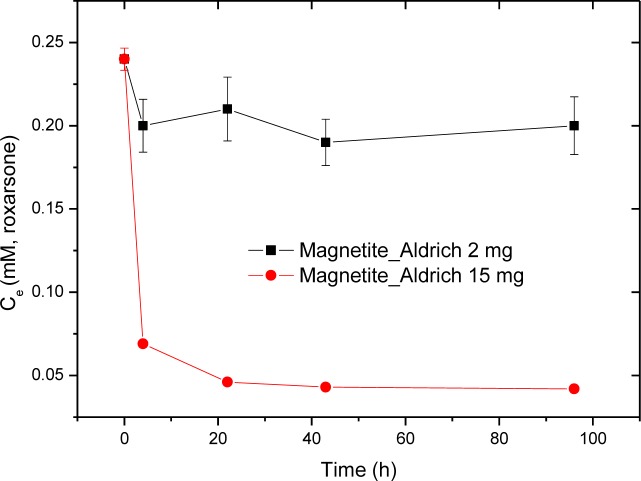
The sorptive equilibration of roxarsone (*C*_o_: 0.24 mM, *V* = 0.020 L) with commercial magnetite (Aldrich) at pH 7.00 in water at 21 °C against time.

The initial experimental conditions employed roxarsone (*C*_o_ = 0.24 mM, *V* = 0.020 L) at pH 7.00 in water at 21.0 °C for 4, 22, 43, 96 h with 200 rpm shaking are shown in Figure 3, where *C*_o_ is the initial concentration of roxarsone. The time required to reach sorptive equilibrium for roxarsone with commercial magnetite (Aldrich) exceeded 20 h. According to Figure 3, an equilibration time used herein for the sorption of roxarsone was 24 h for an adsorbent dosage of 15 mg at these conditions. The use of higher dosages of adsorbent for the isotherm studies minimizes the random errors related to sample weight and residual levels of unbound adsorbate (*C*_e_), as shown by the calibration data in Figure 2b.

**Figure 4.**
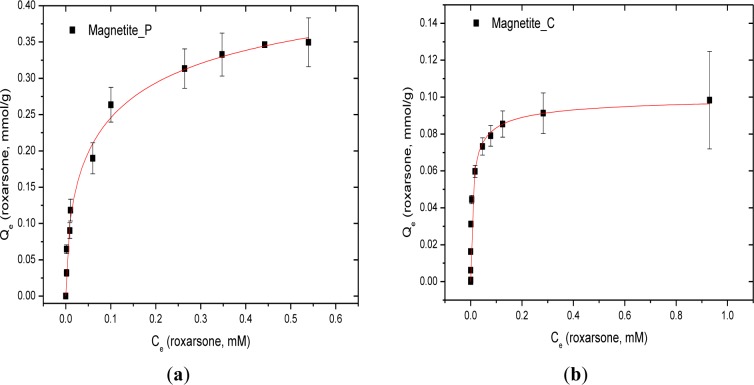

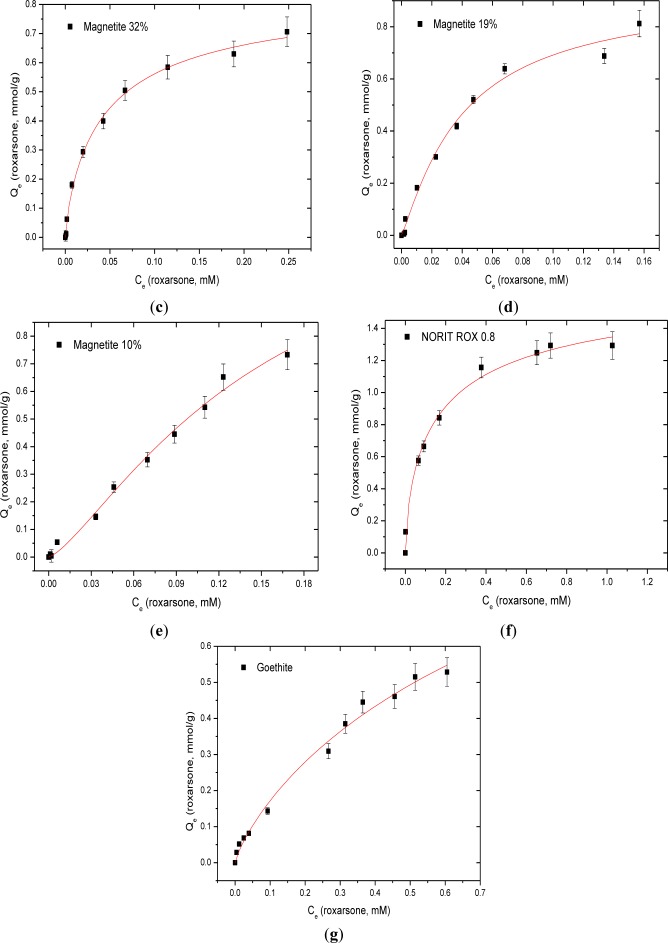
The Sips isotherm fitting results for the adsorption of roxarsone with the Mag-P, Mag-C, CM-32, CM-19, CM-10, GAC, and goethite at pH 7.00 in water at 21 °C for 24 h. (Adsorbent dosage: ~15 mg; *C_o_*: (0.025~1.0 mM); *V* = 0.020 L).

The sorptive uptake results at isotherm conditions for roxarsone with synthetic magnetite (Mag-P), commercial magnetite (Mag-C), GAC-magnetite composites with variable magnetite (32%, 19%, and 10%; w/w%) content, and goethite are shown in Figure 4 and Table 1. The maximum monolayer adsorption capacity of roxarsone (*Q*_m_ = 1.783 mmol/g) was achieved with GAC. Although GAC contains a relatively low intrinsic level of Fe content [17] as an impurity due to its synthetic preparation, the sorptive removal of roxarsone is attributed primarily to noncovalent interactions such π-π stacking and H-bonding between the phenyl ring of roxarsone and the graphene surface of GAC. In the case of composite materials (CM-10, -19, and -32), the *Q*_m_ value of roxarsone increased as the content of the activated carbon content increased, as anticipated for adsorptive processes influenced by the hydrophobic effect. By contrast, the iron oxide components such as magnetite, goethite, and magnetite composites which possess π-electron acceptor (Lewis acid) sites due to the presence of iron species on the adsorbent surface, especially for inorganic arsenate [12,21–23]. As well, favourable Lewis acid-based interactions may contribute to the formation of coordination complexes on the iron oxide surface because complexes between Fe species and the oxygen atoms of the roxarsone anion [25,26]. Adsorption of roxarsone on the heterogeneous surface sites of magnetite composites (CM-10 and CM-19) are supported by the exponent term (*n* > 1) from the Sips isotherm modeling parameters. Mag-C showed Langmuir adsorption (*n* = 1) because of its relatively uniform and homogeneous surface.

The equilibrium uptake (*Q*_e_, mg/g) of an adsorbent toward an adsorbate is calculated using Equation (1). *C*_o_ is the initial concentration (*M*) of the adsorbate, *C*_e_ is the equilibrium concentration (*M*) of the adsorbate, *V* is the volume (L) of the adsorbate, and *m* (g) is dosage level of the adsorbent employed. The monolayer adsorption capacity of roxarsone (*Q*_m_; mmol/g) was obtained using the Sips model [27] using Equation (2), because it accounts for the empirical adsorption results that describe surface heterogeneity or homogeneity, described by the Freundlich or Langmuir isotherm models:

$$Q_{e}=\frac{(C_{o}-C_{e})\times V}{m}$$ (1)

$$Q_{e}=\frac{Q_{m}K_{S}C_{e}^{n}}{1+K_{S}C_{e}^{n}}$$ (2)

*K*_s_ is the Sips equilibrium constant and the exponent term, *n*, describes the sorbent surface heterogeneity that account for multiple adsorption sites. When *n* is very low, Equation (2) reduces to the Freundlich equation. The Sips isotherm model provides a general description of various types of monolayer adsorption, both the Freundlich (*n* > 1) and Langmuir (*n* = 1) models, whilst providing an estimate of *Q*_m_. The Sips model is empirically based and valid only up to certain concentration values. At low concentration, a linear relationship between *Q*_e_ and *C*_e_ occurs [28], as predicted by Equation (2). Heterogeneous adsorption is observed when *n* > 1, as evidenced by multi-site adsorption at surface sites on the adsorbent through the formation of inner-/outer-sphere complexes, π-π stacking, or H-bonding interactions.

**Figure 5.**
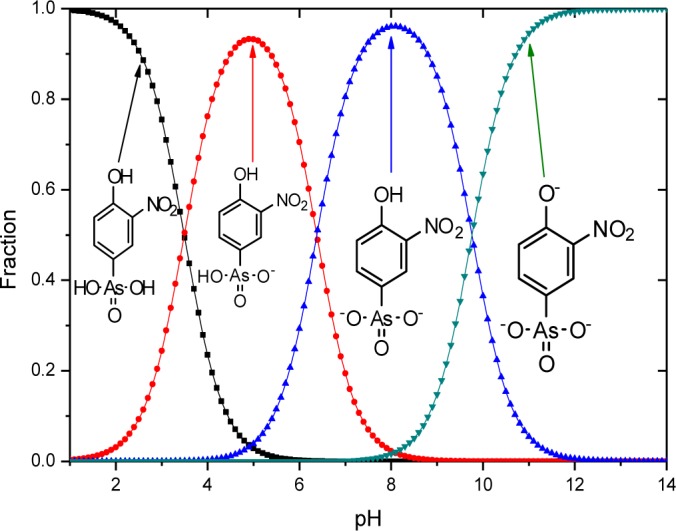
The protolytic speciation of roxarsone in water is expressed as mole fraction against pH.

The sorptive uptake of roxarsone in aqueous solution depends on various factors: pH, buffer system, ionic potential of adsorbate, and pH_pzc_ of adsorbent. The advantage of employing a buffer solution instead of an unbuffered aqueous solution for sorption relates to the ionic strength and constant pH in the case of a buffer. As the concentration of buffer exceeds that of the adsorbate, the activity of the adsorbate approaches unity due to the increased dissolution of the adsorbate by the increased hydronium ion concentration from the buffer resulting in greater dissolution of adsorbate by protolysis [29]. Moreover, controlled speciation of an adsorbate occurs when the pH of the system is maintained. For example, an adsorbate with multiple protonation and oxidation states such as the oxoanions of Se and As (e.g., SeO_3_^2−^, SeO_4_^2−^, AsO_3_^3−^, and AsO_4_^3−^) which may exits various protolytic forms depending on the solution environment during sorption or analysis. In the case of UV-visible absorbance measurements, pH variation may occur due to the hydrolysis of CO_2_ in air, especially in unbuffered aqueous solution. With increasing ionic strength of the buffer system, electrostatic repulsion between an adsorbent and an adsorbate decrease due to the constriction of the electric double layer of the adsorbent as ionic strength increases. Selection of the buffer is important because an ion with the highest ionic potential in solution will interact first with the adsorbent. Therefore, the ionic potential of the buffer should be lower than that of the adsorbate. The ionic potential (ψ) is the ratio of the adsorbate oxidation state number (*z*) over the ionic radius (*r*) of the adsorbate ion (ψ = *z*/*r*); where *r* = 0.6 nm (phthalate), *r* = 0.42 nm (roxarsone, neutral), *r* = 0.45 nm (selenite), and *r* = 0.4 nm (phosphate: H_2_PO_4_^−^ and HPO_4_^2−^). As the ionic potential of the adsorbate increases, stronger interactions occur between the adsorbate and the adsorbent. Another factor affecting the sorption behaviour is the pH because the net surface charge of the adsorbent is zero when the pH matches the pH_pzc_ of the adsorbent. Thus, the pH conditions can be chosen to maximize the electrostatic interaction between adsorbate and adsorbent in solution by accounting for the pK_a_ of the adsorbate and the pH_pzc_ of the adsorbent. If the pH (solution) < pK_a_ (adsorbate) and pH_pzc_ (adsorbent), the surface of the adsorbate and the adsorbent are positively charged. The surface of the adsorbent and adsorbate are negatively charged for the opposite case; pH (solution) > pK_a_ (adsorbate) and pH_pzc_ (adsorbent). At these boundary conditions, sorptive uptake will be negligible due to electrostatic repulsion between the adsorbate/adsorbent systems. At the following condition: pH_pzc_ (adsorbent) < pH (solution) < pK_a_ (adsorbate), the surface charge of the adsorbate is positive, the adsorbent is negative, and the maximum sorptive uptake is possible. At the following condition: pK_a_ (adsorbate) < pH (solution) < pH_pzc_ (adsorbent), the surface charge of the adsorbate is negative, the adsorbent is positive, and maximum sorption uptake is also possible [30]. Roxarsone has three pK_a_ values in aqueous solution and its speciation at variable pH is shown in Figure 5.

The pH at the point of zero charge (pH_pzc_) for magnetite is estimated to be 6.5 [31], while the pK_a_ values of roxarsone are 3.49, 6.38, and 9.76 and 8.40 [32]. The measured pH_pzc_ of GAC (NORIT ROX 0.8) (Norit Americas Inc., Marshall, TX, USA) by mass titration is 7.3 and the literature value of pH_pzc_ of goethite is 3.2 for a mineral type and 6.7–9.0 for the synthetic mineral depending on its relative water content [31]. The use of a buffer at pH 7.00 can produce a net negative surface charge for magnetite, but it may be positive for GAC-based composites depending on the iron oxide composition. At pH 7, the ionic charges of roxarsone at this pH may afford a combination of species; mono-anion (20%) and di-anion (80%), as shown in Figure 5. Therefore, favorable electrostatic interactions are anticipated between magnetite composites, GAC, and goethite with roxarsone at these conditions. H-bonding and van der Waals interactions occur between roxarsone and each of the adsorbents. By comparison with inorganic arsenate, the roxarsone mono-anion may coordinate in a monodentate-mononuclear fashion with iron oxide species [33] situated in the interstitial void area of the tetrahedral (Fe^2+^ and Fe^3+^) and octahedral (Fe^2+^) sites of the magnetite inverse spinel structure [34] to form an inner-sphere complex [25]. Similarly, the roxarsone di-anion may be coordinated as a bidentate-binuclear or a bidentate-mononuclear complex with Fe species. Besides these surface complexes, there may be different configurations of surface bound complexes such as an outer-sphere ion-pair adsorption complex or a solid solution of the roxarsone in the oxide phase [25]. Various types of surface complexes have been reported elsewhere [35,36] using Extended X*-*ray Absorption Fine Structure (EXAFS) and Attenuated Total Reflectance Fourier Transform Infrared Spectroscopy (ATR*-*FTIR). The formation of similar surface complexes between magnetite, magnetite composites, GAC, and goethite with the roxarsone anion species are consistent with the foregoing possibilities. The sorptive interaction of roxarsone with various adsorbents in this study was attributed to several possible interactions: (*i*) π-π interaction between the π-electron rich graphene rings of GAC and theπ-electron deficient phenyl ring of roxarsone [37,38], (*ii*) surface complexation between the arsenate group of roxarsone and iron oxide species, and (*iii*) H-bonding. The ionic potential of roxarsone at pH = 7.00 was 4.8 for the di-anion (80%) and 2.4 for mono-anion (20%) species, while that for phosphate monoanion (H_2_PO_4_^−^) was 2.5, and the ionic potential for phosphate (PO_4_^3−^) is 7.5. Therefore, the sorptive uptake of roxarsone is favored over H_2_PO_4_^−^, in agreement with independent experimental results [37].

### 2.2. Sorption Kinetics

**Figure 6.**
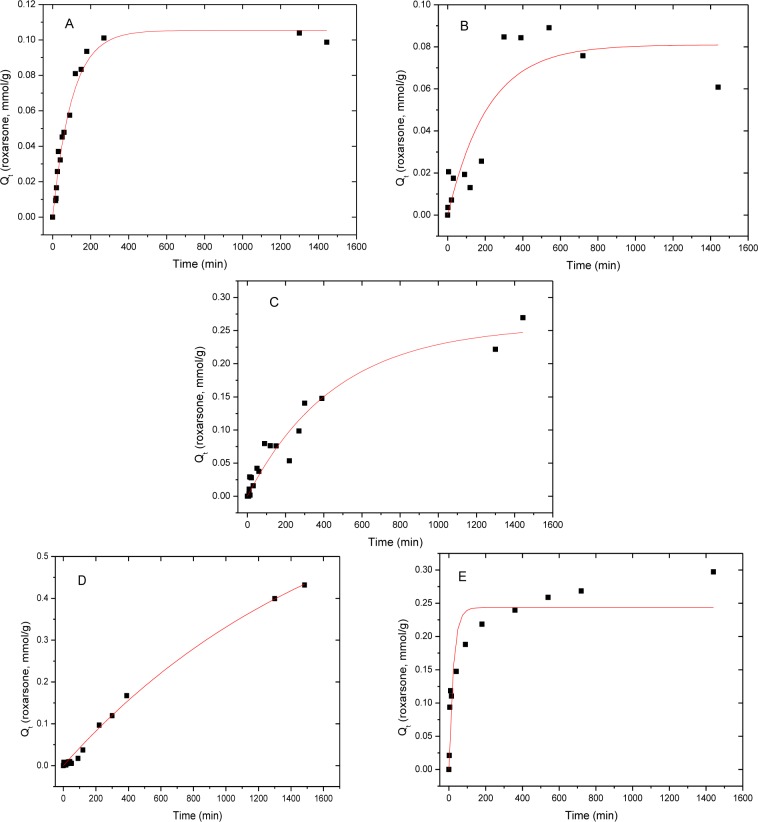
The kinetic uptake of roxarsone (*Q_t_*, mmol/g) with various adsorbents ((**A**): Mag-P, (**B**): Mag-C, (**C**): CM-19, (**D**): GAC, (**E**): goethite), (*C*_o_: 0.18 mM, amount of adsorbents: 0.030 g, *V* = 0.20 L) at 21 °C at pH 7.00 in water at variable time intervals.

The sorption kinetics and parameters for roxarsone (*Q*_e_, mmol/g) with various sorbent materials are shown in Figure 6 and in Table 2. The rate constant, *k,* was determined using the pseudo-first-order (PFO) model (Equation (3)) [39] and the pseudo-second-order (PSO) model (Equation (4)) [40–42]. Goethite showed a higher *R*^2^ value (0.938) with the PSO model, relative to the *R*^2^ value (0.871) for the PFO model. Therefore, the PFO analysis was estimated for all of the adsorbents. The parameter *q*_t_ (mg/g) is the adsorbed amount of an adsorbate at time *t*, *q*_e_ (mg/g) is the adsorbed amount of the adsorbate at equilibrium, while *k*_1_ and *k*_2_ are the rate constants (min^−1^; PFO and g mg^−1^ min^−1^; PSO). Integration of Equations (3) and (4) at the boundary conditions (*q_t_* = 0 at *t* = 0 and *q_t_* = *q_t_* at *t* = *t*) with rearrangement yields the non-linear PFO (Equation (5)) and the non-linear PSO (Equation (6)), respectively:

$$\frac{dq_{t}}{dt}=k_{1}(q_{e}-q_{t})$$ (3)

$$\frac{dq_{t}}{dt}=k_{2}{\left(q_{e}-q_{t}\right)}^{2}$$ (4)

qt=qe(1−e−k 1t) (5)

$$q_{t}=\frac{k_{2}q_{e}^{2}t}{1+k_{2}q_{e}t}$$ (6)

To obtain the rate constant, PFO model was used because root mean square error (RMSE) of the PFO model was better than that of PSO model (*cf*. Table 3), with the exception of goethite. Goethite showed better agreement with the PSO model; however, the values of *Q*_e_ (mmol/g) were similar for the PFO and PSO models, as shown in Table 2.

As shown in Table 2, GAC showed the highest sorptive removal (*q*_e_) of roxarsone in spite having the lowest observed rate constant (*k*_obs_). The *q*_e_ values for the iron oxide materials and its composites are greater, as follows: CM-19 ≈ goethite > Mag-P > Mag-C. The trends are related to the various intermolecular interactions between the adsorbate and roxarsone, in addition to the hydration of each species. GAC has a large graphene surface area (951 m^2^/g) which may preferentially adsorb roxarsone due to electron donor-acceptor (EDA, π-π) interactions, H-bonding, and hydrophobic effects. CM-19 has a relatively large surface area (754 m^2^/g) and likely adsorbs roxarsone through similar interactions observed for GAC, as well as inner-sphere surface complexation. The slight attentuation in *q_e_* values may be due to pore blockage of GAC due to iron oxide species and/or the reduced binding affinity of iron oxide sites. Goethite has a reduced surface area (214 m^2^/g) relative to CM-19; however, the presence of hydroxyl groups (Fe-OH) contribute to enhanced binding of roxarsone via H-bonding and inner-sphere surface complexation. The reduced surface areas and uptake of roxarsone by Mag-P (94 m^2^/g) and Mag-C (41 m^2^/g) are similar to that obtained for CM-19. The attenuated *q_e_* values may be due to the decrease in surface area and the surface reactivity of available Lewis acid species of Mag-C, as described above. The uptake of roxarsone is correlated with the surface area of GAC and its composite materials, and this is attributed to the favourable interaction of the phenyl ring of the adsorbate with the graphene units of the adsorbent, in accordance with hydrophobic effects. Although the uptake of inorganic arsenate was not measured herein, it is anticipated that the *q*_e_ values correlate with the iron oxide content, according to studies reported elsewhere [12,21–23]. However, the kinetic results in Table 2 (*k*_obs_) were related to the relative polarity of the adsorbent surface since composites and iron oxide minerals display higher values of *k*_obs_. The relationship between *k*_obs_ and hydration phenomena is antcipated due to the polar nature of roxarsone and the relevance of various steps in the overall adsorptive process (e.g., film and pore diffusion). The polarity of adsorbents decreased by the order of goethite > Mag-P > Mag-C > CM-19 > GAC. Therefore, goethite could diffuse through the boundary layer fastest according to the largest rate constant. As the magnetite content decreased, the rate constant also decreased relative to a decreasing polarity of the adsorbent surface. Mag-P was more polar than that of Mag-C because the surface area differed by a factor of two, which probably accomodates more Fe species at the tetrahedral and octahedral sites.

Various adsorbent materials have been used for uptake of organo-arsenicals [As (V); mg/g] such as roxarsone from other independent studies, and these results are summarized in Table 4 on the basis of As (V) content. In the case of secondary binding between the arsenate anion moiety of roxarsone and the iron oxide sites of the adsorbent, adsorption is thermodynamically favored as a bidentate-binuclear inner-sphere surface complex on the surfaces of magnetite composites [43] and goethite. As discussed in Section 2.1, the sorptive interaction of roxarsone with various adsorbents was attributed to EDA, surface complexation, and H-bonding. Considering the ionic potentials of the buffering agent and the adsorbate, uptake is favoured with roxarsone, not dihydrogen phosphate. Moreover, the measurement of absorbance of roxarsone was done at specific time intervals and the *q*_e_ (mmol/g) parameter is the same regardless of the adsorbent even if dihydrogen phosphate was the major adsorbate species of interest.

**Scheme 1.**
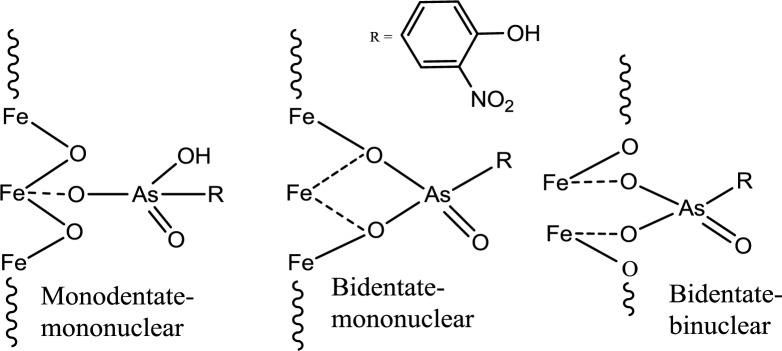
Secondary adsorption of roxarsone onto the surface of an iron oxide/oxyhydrate at pH 7.00 in water (adapted from [12,36]).

**Scheme 2.**
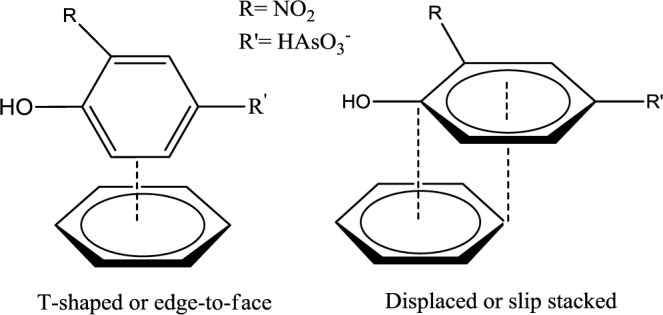
The primary adsorption mechanism of roxarsone onto the graphene surface of NORIT ROX 0.8 via a π-π stacking mechanism at pH 7.00 in water (adapted from [38]).

The monodentate-mononuclear inner-sphere surface complex is regarded as the minor contributor by recalling the anion speciation at the pH conditions employed herein, as illustrated in Figure 5. The adsorption mechanism for the arsenate moiety of roxarsone is proposed in Scheme 1, and is anticipated to be more important for inorganic forms of arsenic or its composite materials with greater loading of iron oxide on the graphene surface. In the case of magnetite materials, roxarsone is adsorbed as an outer-sphere surface complex due to the electrostatic repulsion between magnetite and roxarsone. H-bonding may occur between the surface hydroxyl groups of magnetite with the roxarsone anion or bound water which offsets electrostatic repulsive interactions. In the case of GAC, π-π displaced or T-shaped stacking interactions between the phenyl group of roxarsone and the graphene surface are the dominant interactions (*cf*. Scheme 2) [38]. Additional H-bonding and inner-sphere surface complexation may occur; however, the pheny ring interactions are considered as the main driving force for the uptake of roxarsone by GAC and its composite materials, in agreement with the hydrophobic effect. Considering the adsorptive rate constant values (*k*_obs_) in Table 2, the bidentate-binuclear inner-sphere surface complex with goethite may display a greater degree of ligand exchange; whereas, magnetite composites and GAC may undergo slower ligand exchange through π-π interactions affording enhanced sorptive interactions. Mag-P and Mag-C may be exposed to the slow inner-sphere surface complex formation rate and H-bonding where steric effects due to hydration processes attenuate the value of *k*_obs_. When the roxarsone anion is adsorbed onto the surface of iron oxide/oxyhydrate, a modified triple-layer model (TLM) may provide an understanding of the secondary importance of such surface complexation processes [46]. In this model, the adsorbed anion may be bound to the α-layer (equivalent to the inner Helmholtz plane), forming an inner-sphere complex, and the β-layer (equivalent to the outer Helmholtz plane), forms an outer-sphere surface complex [46].

## 3. Experimental Section

### 3.1. Synthesis and Experimental Conditions

**Figure 7.**
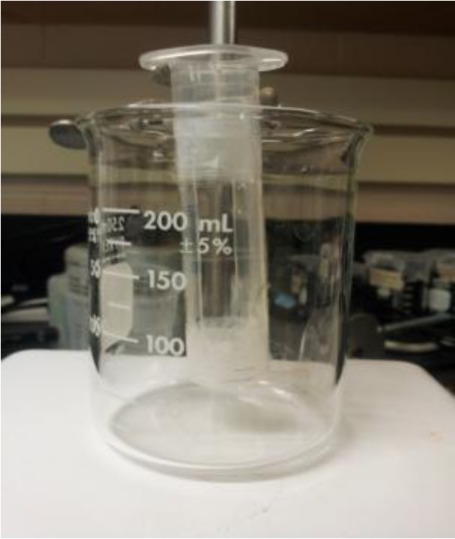
Experimental set-up for *in-situ* kinetic uptake studies.

Roxarsone was obtained from Haohua Industry Co. Ltd. (Jinan, China) was purified by recrystallization from water [37]. Briefly, 1 g of roxarsone was dissolved in 25 mL of Millipore water at 65 °C with stirring and this solution was hot filtered through Whatman No.2 filter paper at ambient conditions. The filtrate solution was allowed to cool slowly before being placed in a refrigerator for 24 h. Aggregates of small orange crystals were collected and isolated through filtration with drying at 50 °C for 1 h to afford a light tan powder product. Synthetic magnetite (Mag-P) was prepared by co-precipitation methods, as described elsewhere [47]. Magnetite composites with activated carbon were prepared by the same method, but the slurry of activated carbon solution (200 mL) containing 1.0 g of activated carbon was prepared before adding Fe^3+^/Fe^2+^ (2:1 molar ratio). The detailed experimental conditions are given in Table 4 and it should be noted that all sorption experiments were carried out in water with no phosphate buffer. Absorbance of the sample solutions were measured with a UV-vis spectrophotometer (Varian Cary 100 SCAN, Agilent Technologies Inc., Santa Clara, CA, USA) at λ = 244 nm with a quartz cuvette where the aqueous samples were diluted in phosphate buffer at pH 7.00. Each adsorbent has a notable magnetic susceptibility except the GAC and goethite; thus, kinetic experiments were performed using an aluminum stirring paddle with an overhead mixer with a semi-permeable dialysis tubing (Sigma-Aldrich Canada, Ltd., Oakville, Canada, molecular weight cut-off 12,000–14,000 *amu*), as shown in Figure 7. The dialysis tube was cut to size and applied as a cover for an open-ended syringe body with parafilm, as shown in Figure 7.

### 3.2. Equilibrium Sorption Studies

A stock solution (1.00 mM) of the purified roxarsone in Millipore water was prepared at ambient pH, which was further diluted in Millipore water to provide working solutions of roxarsone (*C*_o_: 0.026, 0.050, 0.076, 0.100, 0.200, 0.300, 0.500, 0.600, 0.700, 0.800, 0.900, 1.00 mM). Twenty mL of the working solutions were added into 50 mL centrifuge tubes and placed in a SCILOGEX SK-0330-Pro rotoshaker (SCILOGEX, LLC, Berlin, CT, USA) operating at 200 rpm for 24 h. After reaching equilibrium, the samples were centrifuged with a Beckman Coulter Avanti J-E Centrifuge (Beckman Coulter, Inc., Indianapolis, IN, USA) for 30 min. at 25,000 rpm; 10 mL of the supernatant was carefully transferred to a sample vial for subsequent analysis. UV-Vis measurements were carried out using 1.0 mL of supernatant solution in a quartz cuvette where further dilutions were made using 1.0 mL of 10 mM KH_2_PO_4_ buffer (pH = 7.00 ± 0.02).

### 3.3. Kinetic Uptake Studies

Two hundred mL of 0.18 mM roxarsone in Millipore water was added into a 250 mL beaker. The syringe wrapped with the dialysis tubing was introduced in the solution and soaked for 20 min to reach equilibrium. Then, 0.030 g of each adsorbent was added into the beaker while the solution was stirred at 510 ± 10 rpm. Aliquots of 0.30 mL were taken at the scheduled time *t* and absorbance was measured at 244 nm. The aliquots were diluted with 2.70 mL of 10 mM KH_2_PO_4_ buffer (pH 7.00 ± 0.02) prior to UV-vis analysis.

## 4. Conclusions

Equilibrium and kinetic uptake studies in water for various adsorbents were conducted and the results revealed that NORIT ROX 0.8 (GAC) provided the most favorable overall adsorption, followed by goethite, CM-10, CM-19, CM-32, Mag-P, and Mag-C. This result showed that the high surface area of GAC afforded efficient removal of roxarsone via favorable π-π interactions between the roxarsone phenyl moiety and the graphene surface (*cf*. Scheme 2). The decreasing sorptive uptake paralleled a decrease in the content of GAC for the composite materials. Thus, the Lewis acid-base interactions of the arsenate anion with the iron oxide surface sites were considered secondary in nature for roxarsone relative to those depicted in Scheme 2. However, the sorptive removal of roxarsone with goethite was pronounced because of its polar nature due to the surface hydroxyl groups of this mineral surface and the propensity for H-bonding interactions. Secondary surface complexes between the roxarsone anion and the iron oxide/oxyhydrate surface sites may adopt one or more mechanisms: inner-sphere of monodentate-mononuclear, bidentate-mononuclear, and bidentate-binuclear complexes. The secondary interactions (*cf*. Scheme 1) described above are anticipated to be more important for inorganic forms of arsenate with iron oxide mineral surfaces. The details of these surface adsorption processes are the subject of future studies.

## Figures and Tables

**Table 1.** Sips isotherm parameters for roxarsone with various adsorbents at pH 7.00 in water at 21 °C. (Adsorbent dosage: ~15 mg; *C*_o_: (0.025~1.0 mM); *V* = 0.020 L).

Adsorbents	*Q*_m_ (mmol/g)	*K_s_* (L/g)	*n*	*R*^2^	Chi^2^/DoF
Mag-P	0.500 ± 0.083	3.50 ± 2.00	0.559 ± 0.086	0.991	2.00 × 10^−4^
Mag-C	0.102 ± 0.008	19.3 ± 13.5	0.624 ± 0.104	0.977	4.00 × 10^−5^
Mag-32	0.862 ± 0.066	12.1 ± 5.0	0.812 ± 0.078	0.996	3.40 × 10^−4^
Mag-19	0.936 ± 0.096	39.7 ± 32.6	1.15 ± 0.18	0.990	1.13 × 10^−3^
Mag-10	1.39 ± 0.45	12.9 ± 14.0	1.34 ± 0.25	0.993	6.30 × 10^−4^
GAC	1.78 ± 0.27	3.05 ± 1.70	0.662 ± 0.132	0.995	1.78 × 10^−3^
Goethite	1.59 ± 1.00	0.792 ± 0.789	0.815 ± 0.135	0.992	4.20 × 10^−4^

Note: Chi^2^/DoF (degree of freedom) is a diagnostic goodness-of-fit parameter.

**Table 2.** The kinetic (PFO) sorption parameters for roxarsone (*Q*_e_, mmol/g) with the various sorbent materials (*C*_o_: 0.18 mM, amount of adsorbents: 0.030 g, *V* = 0.20 L) at 21 °C at pH 7.00 in water for 24 h.

Adsorbents	*q*_e_ (mmol/g)	*k*_obs_ (min^−1^) × 10^3^	*R*^2^	Chi^2^/DoF
Mag-P	0.105 ± 0.007	9.86 ± 1.58	0.932	1.20 × 10^−4^
Mag-C	0.081 ± 0.011	4.68 ± 1.86	0.803	2.60 × 10^−4^
CM-19	0.258 ± 0.018	2.18 ± 0.33	0.940	3.60 × 10^−4^
GAC	0.748 ± 0.091	0.580 ± 0.100	0.995	1.10 × 10^−4^
Goethite	0.244 ± 0.015	41.5 ± 12.3	0.871	1.34 × 10^−3^
Goethite ^1^	0.262 ± 0.013	210 ± 60.0	0.938	6.50 × 10^−4^

Note: Mag-P is the synthetic material, Mag-C is the commercial material.^1.^ Represents the estimate obtained using the PSO model (Equation (3)).

**Table 3.** Comparison of root mean square error (RMSE) values of pseudo-first-order (PFO) and pseudo-second-order (PSO) models for roxarsone adsorption with various adsorbents at 21 °C at pH 7.00 in water at variable time intervals.

Adsorbents	PFO	PSO
Mag-P	4.76 × 10^−4^	7.95 × 10^−3^
Mag-C	4.55 × 10^−2^	4.50 × 10^−2^
CM-19	3.97 × 10^−1^	3.97 × 10^−1^
GAC	9.93 × 10^−3^	1.01 × 10^−2^
Goethite	3.50 × 10^−2^	2.44 × 10^−2^

**Table 4.** Various adsorbents and their sorptive removal of roxarsone calculated according to As^5+^ content.

Adsorbent Material	Conditions (*C*_o_ in [As^5+^])	Uptake (As^5+^, mg/g)	Reference
Multi-walled carbon nanotubes (MWCNTs)	pH 2–12; 10 °C; *C*_o_: 50 mL of 5–40 ppm (equilibrium), 10–40 ppm (kinetic); 100 mg adsorbent	3.65–3.85 (equilibrium) 0.997–2.88 (kinetics)	[44]
Goethite	pH 5–9; 23 °C; 24 h; *C*_o_: 3.7 ppm; 45 mg adsoebent /L	0.283–0.0883 (kinetics)	[45]
Mag-P	Equilibrium pH 7.0 buffer; 24 h; 21 °C; *C*_o_: 20 mL of 6.56–263 ppm; 15 mg adsorbent Kinetic pH 7.0 buffer; 24 h; 21 °C; *C*_o_: 200 mL of 47.3 ppm; 30 mg adsorbent	37.5 (equilibrium) 8.24 (kinetics)	This study
Mag-C	–	8.39 (equilibrium) 6.07 (kinetics)	–
CM-32	–	64.6 (equilibrium)	–
CM-19	–	72.4 (equilibrium) 19.3 (kinetics)	–
NORIT ROX 0.8 (GAC)	–	134 (equilibrium) 56.1 (kinetics)	–
Goethite	–	119 (equilibrium) 18.2 (kinetics)	–

**Table 4.** The experimental conditions for the uptake studies of roxarsone with various adsorbents at pH 7.00 in water for 24 h.

Sorption	Adsorbents	*T* (°C)	Amount (mg)	*C*_o_ (mM)	Volume (L)
Equilibrium	Mag-P	21	15	0.025–1.00	0.020
	Mag-C	21	15	0.025–1.00	0.020
	CM-32	21	15	0.025–1.00	0.020
	CM-19	21	15	0.025–1.00	0.020
	CM-10	21	15	0.025–1.00	0.020
	GAC	21	15	0.025–1.00	0.020
	Goethite	21	15	0.025–1.00	0.020

Kinetics	Mag-P	21	30	0.18	0.20
	Mag-C	21	30	0.18	0.20
	CM-19	21	30	0.18	0.20
	GAC	21	30	0.18	0.20
	Goethite	21	30	0.18	0.20
